# Prevalence, perceived risk and associated factors of tobacco use amongst young, middle-aged and older adults: analysis of a national survey in Jamaica

**DOI:** 10.11604/pamj.2022.43.185.36517

**Published:** 2022-12-08

**Authors:** Joni-Gaye McLeary, Geoffrey Walcott, Wendel Abel, Gabrielle Mitchell, Kunal Lalwani

**Affiliations:** 1 Department of Community Health and Psychiatry, University of the West Indies, Mona, Jamaica

**Keywords:** Tobacco, Jamaica, household survey, prevalence, risk perception, adults

## Abstract

**Introduction:**

tobacco smoking remains a significant threat to public health. This paper sought to examine the prevalence, perceived risk and the associated factors of current tobacco use amongst Jamaican adults.

**Methods:**

this study is a secondary data analysis of the Jamaican National Household Survey 2016. The data for this study was extracted from a pre-coded questionnaire using a standardized extraction sheet. Adults were categorized into young adults (18-35), Middle-aged adults (36-55) and Older adults (56 and older) groups. Statistical analysis was performed to determine the prevalence, perceived risk and associated factors of tobacco use among Jamaican adults.

**Results:**

young adults had the lowest lifetime prevalence of tobacco smoking at 23% yet the highest percentage of current users at 48.3% (p=0.000), with gender being the only significant associated factor. Males were 2.565 times more likely to be current tobacco users than females (p < 0.01). In middle-aged adults, and older adults the highest predictive factor was risk perception. Current tobacco use was 3.375 times higher in middle-aged adults (p=0.044) and 2.815 times higher in older adults with low risk perception (p=0.025).

**Conclusion:**

four out of 10 Jamaican adult tobacco users are current users. Young adults had significantly fewer mitigating factors to tobacco usage despite being the most prevalent group for current use. Significantly, perception of risk impacted current usage in middle-aged and older adults but not in younger adults. Innovative and targeted approaches are needed in young adults such as incorporating the health and wellness promotion model with tobacco emphasis.

## Introduction

Tobacco use refers to persistent consumption of the tobacco plant “Nicotiana” and its products [1]. Whether smoked, chewed or through secondary exposure, tobacco use is foremost deleterious [2,3], resulting in significant morbidity and mortality [4], and represents the most substantial risk factor unique to major non-communicable diseases (NCD) [5,6]. Globally, tobacco smoking had the highest substance-attributable mortality rate and the highest disability-adjusted life years (DALY) when compared to alcohol, marijuana, and other illicit drugs [7]. Additionally, tobacco smoking increases the risk for immunological issues, ophthalmological disease, male infertility, erectile dysfunction, female infertility and complicated ectopic pregnancies demonstrating that its effect is multi-systemic [8]. Significantly, tobacco smoking contributes to most lung cancer and associated deaths in both sexes over the last four decades [3]. A smoker´s lifespan is reduced by ten years when compared to non-smokers which highlights the severe impact it has on the individual´s health and longevity [9]. Moreover, studies indicate that most individuals who want to quit, find it difficult to do so [10,11], underscoring the overwhelming challenge smokers face to abstain from tobacco in any of its forms [3].

Tobacco use has been an ongoing health concern in the country of Jamaica. Jamaicans spend nearly 40% of their yearly income on tobacco-related products, with a significant number of persons diagnosed with chronic obstructive pulmonary disease and lung cancers, spending greater than 50% of their yearly income treating their disease [12]. As such, tobacco smoking carries an economic burden for both smoker and the country. Due to the alarming impact on the health and wellbeing of the citizens, Jamaica signed on to the World Health Organization Framework Convention on Tobacco Control (WHOFTC) in 2005. Various interventions have been made by the Jamaican government which includes more graphic health warning depictions on cigarette packaging display surfaces, restricting smoking in public spaces and work environments, banning of tobacco advertising on radio and television, and increased taxation on tobacco products. Presently, tobacco legislation, health promotion, and education have been the main thrust in reducing tobacco smoking [13]. Notwithstanding, according to the Pan American Health Organization´s (PAHO) most recent report, Jamaica had the second highest current tobacco prevalence in the Caribbean at approximately 17% amongst adults [14]. This further highlights the fact that Jamaica still experiences a high disease burden, likely attributable to the individual´s choice or willingness to smoke and/or as a result of dependence. A number of noteworthy sociodemographic factors serve as determinants for the initiation and continued use of tobacco. The World Health Organization (WHO) reports the global lifetime prevalence of tobacco gradually declining in each age group, with lowest seen in young adults less than 35 years and the highest prevalence within the middle-age group at ages 45-54 in both sexes [15]. Adult studies on tobacco smoking in Latin America and the Caribbean are found within multinational surveys and analyses [16,17]. Latin American countries comparatively, tend to have a higher prevalence of tobacco use likely attributable to extensive tobacco cultivation [16]. However, there is a dearth of data on the prevalence distribution of tobacco use amongst the young adult, middle-aged, and older adult population groups in Jamaica.

There is a higher rate of consumption amongst males regardless of age, both globally and regionally [17,18]. Literature suggests that men are fueled to smoke by the effects of the reward pathway [19], whereas loneliness, depressive symptoms, and the added weight controlling benefit potentiates smoking in women [20]. Notwithstanding, social connectivity and support systems derived within marriage can be a protective factor against tobacco smoking and contribute significantly to decreasing psychological distress [21,22]. The majority of studies have collectively highlighted that acquisition and increasing levels of education, predispose to lower prevalence of tobacco smoking, likely due to acknowledging the harmful effects of smoking [23,24]. There is conflicting evidence regarding employment and prevalence rates of tobacco smoking, with a number of studies suggesting higher prevalence amongst those who were unemployed in developed nations [25-27]. This contrasts with a higher prevalence of tobacco smoking observed amongst employed persons in middle and lower-income countries [28], likely as a result of job strain and work-related stress [29]. In furtherance, studies have shown a higher prevalence of tobacco use in persons with laborious, physical jobs, such as construction in comparison to professionals [30,31].

Peer-reviewed literature reveals that despite smokers´ awareness of the associated health risks of smoking, they tend to have a lower perceived risk of addiction and health consequences associated with tobacco use, than non-smokers and former smokers. This therefore increases the likelihood of continued use while making it is less likely to contemplate quitting [32,33]. In Jamaica there is a paucity of literature on the risk perception of tobacco use amongst the young adult, middle-aged, and older adult population groups. Most studies analyze tobacco use as if prevalence and associated factors are collectively the same across the entire population sample. This identifies a methodological gap in the literature that this study is geared towards addressing. This level of analysis has not been previously done and will provide greater insight for policymakers into the identification, and implementation of appropriate interventions, needed to reduce the prevalence and disease burden of tobacco use within the different adult groups in Jamaica. The current study examined tobacco use amongst a nationally representative sample of young adult, middle-aged, and older adult groups in the Jamaican population to 1) determine the prevalence of current tobacco use; 2) elucidate the perceived risk associated with tobacco use; and 3) identify the associated factors of current tobacco use.

## Methods

**Study design:** the initial study was a cross-sectional survey of a national population sample investigating the prevalence and patterns of drug use amongst Jamaicans [34]. This study is a secondary data analysis of the National Household Survey, Jamaica 2016. The Ministry of National Security granted approval for the National Drug Use Prevalence Survey to be conducted. Ethical approval for the current secondary analysis study was granted through the Ethics committee of The University of the West Indies, Mona Campus.

**Setting:** the National Drug Prevalence Survey 2016 was designed to focus on the patterns and prevalence of substance use among 12-65 year-olds in Jamaica. Data was collected between April and July 2016 targeting 357 households per parish, totaling a national representative sample of 4,623 households/dwellings. The data was collected from a standardized questionnaire developed by the Inter-American Drug Abuse Control Commission (CICAD) and the Inter-American Observatory on Drugs (OID) and conducted through partnership with Jamaica´s National Council on Drug Abuse (NCDA).

**Participants:** the island of Jamaica comprises 14 parishes and 5,771 Enumeration Districts (EDs), which are the smallest geographic units that allow for collection of survey data. The survey was conducted using a stratified multi-stage cluster sampling method of which the primary sampling clusters were the enumeration districts (ED). Sampling weights were calculated by the probability of selection and non-response weights. Post-stratification weights were applied to ensure that the distribution of the weighted sample matched the population. In the initial survey, the inclusion criterion for participation was that all respondents had to be between the age of 12 and 65, and residing in Jamaica for at least 6 months [34]. The inclusion criterion for this secondary analysis study was all respondents between the ages 18-65. The exclusion criteria were for persons aged 17 and below. This study contained no identifying data of respondents, and no direct or indirect contact was made with any respondents.

**Study size:** there were 22 EDs in each parish with Sixteen (16) households per ED. The first dwelling within each ED to be interviewed was selected randomly, and one individual aged 12-65 years old was chosen by the Kish methodology to be the interviewee. The total sample for the initial survey was 4,623 individuals. This study is a secondary data analysis of the National Household Survey, Jamaica 2016. The sample analysed in this study was a subset of 4079 adults aged 18-65, extracted from the original study sample. Adults were categorised into groups consisting of Young Adults (YA), Middle-aged Adults (MA) and Older Adults (OA) with age ranges of 18-35, 36-55, and 56 and older respectively, as suggested by Petry [35].

**Variables:** sociodemographic and associated factors were extracted from a pre-coded questionnaire using a standardized extraction sheet, and variables recoded to facilitate data analysis for correlations and predictive power. The variables included in this secondary analysis were age, gender, marital status, educational attainment, employment, occupational description and risk perception.

**Statistical analysis:** univariate analysis of prevalence data was done showing prevalence percentages, and multivariate analyses done using the Kruskal-Wallis test. Predictive tests were done to identify risk and protective factors using logistic regression analyses. Statistical Package for the Social Sciences (SPDD) version 22 was used for all analyses and statistical significance was determined at p < 0.05 with a confidence interval of 95%. The data were presented in the form of tables and text.

**Data sources/measurement:** for this study, current use was defined as the smoking of tobacco within 30 days preceding the interview, regardless of quantity or frequency, and non-current use as the smoking of tobacco greater than 30 days preceding the interview, regardless of frequency or quantity. Lifetime prevalence was defined as the percentage of individuals in the population who at some point in their lives smoked tobacco, preceding the date of interview. In addition, daily use refers to smoking every day for the past 30 days, whereas less than daily use refers to not smoking tobacco every day for the past 30 days. Risk perception was measured by asking respondents to indicate their level of perceived risk with the question, “In your opinion, please indicate the risk of smoking cigarettes sometimes”, and/or “smoking cigarettes often”. Responses were 1=No risk, 2=Low risk, 3=Moderate risk, 4=High risk. For this study, No risk and Low risk were recoded as 1 to represent Low risk; and Moderate risk and High risk were recoded as 2 to represent Moderate to High risk.

## Results

The population sample of 4079 adults selected in the country of Jamaica with a total population of adults being 2.147 million, represents a confidence level of 99%, and gives a CI of +/- 2.02. Total population sample comprised of 1,798 men and 2,281 women, with the breakdown of adult groups into young adults being 44.1% (n=1800), middle-aged adults being 42.1% (n=1716), and older adults being 13.8% (n=563).

**Sociodemographic characteristics of study respondents:** the majority of tobacco users across the 3 age groups were predominantly male gender compared to females in all age groups. The highest educational attainment was at the secondary level for the majority of tobacco users. Tobacco users with secondary education were highest within each age group and across all age groups. Older adults had the highest percentage of primary education (14.9%) across all age groups. Employed persons especially skilled workers, had the highest percentage across all age groups. The majority of respondents were single (62.4%) (Table 1).

**Table 1:** sociodemographic characteristics of study respondents

Demographics	Young (n=416)	Middle-aged (n=515)	Older (n=210)	Total N=1141
**Gender**				
Male	288	355	162	805(70.6%)
Female	128	160	48	336(29.4%)
**Marital status**				
Married	103	185	101	389(34.1%)
Single	310	311	91	712(62.4%)
Divorced	3	19	18	40(3.5%)
**Education**				
None	3	2	2	7(0.6%)
Primary	17	78	82	177(15.5%)
Secondary	307	369	111	787(69.0%)
Post-Secondary	89	66	15	170(14.9%)
**Employment**				
Employed	246	404	144	794(69.6%)
Unemployed	170	111	66	347(30.4%)
**Occupation Description**				
Professional	5	16	3	24(2.1%)
Highly skilled	81	86	27	194(17.0%)
Skilled	294	345	156	795(69.7%)
Unskilled	30	65	22	117(10.3%)
Military	6	3	2	11(1.0%)

**Lifetime prevalence of tobacco use in adults:** lifetime prevalence of tobacco usage of the total adult sample (N=4079) was calculated at 28% (n=1141), with a gender breakdown of 29.4% women (n=336) and 70.6% men (n=805). The respective lifetime prevalence within each age group was calculated: young adults, middle-aged and older adults were 23% (n=416), 30% (n=515) and 37% (n=210) respectively (Table 2).

**Table 2:** lifetime prevalence of tobacco use in adults

N=4079	Young	Middle-aged	Older	Total
**No**	1384 (77%)	1201 (70%)	353 (63%)	2938 (72%)
**Yes**	416 (23%)	515 (30%)	210 (37%)	1141 (28%)

**Current tobacco use by age cohorts:** within the population sample of tobacco users (N=1141), 41.5% (n=473) were current users, while 58.5% (n=668) were non-current users. The young adult group had the highest percentage of current users at 48.3% (n=201) compared to the middle-aged group at 39.6% (n=204) and older adults which had the lowest percentage of current users at 32.4% (n=68). Kruskal-Wallis test highlighted the older adult age group as having significantly higher means than the middle and young adult age groups for non-current tobacco use (mean ranks=622.78, 579.24 and 527.56 respectively). This was statistically significant (p=0.000) (Table 3).

**Table 3:** current tobacco use by age cohorts

N=1141	Young (n=416)	Middle-aged (n=515)	Older (n=210)	Total
**Current**	201 (48.3%)	204 (39.6%)	68 (32.4%)	473 (41.5%)
**Non-current**	215 (51.7%)	311 (60.4%)	142 (67.6%)	668 (58.5%)
**Mean Rank**	527.56	579.24	622.78	

Kruskal Wallis Test X2 =17.480, df=2, p=0.000

**Current tobacco use patterns by age cohorts:** within the population sample of current tobacco users (N=473), the middle-aged and older adult groups had the higher percentage of daily use at 73.0% (n=149) and 70.6% (n=48) respectively, compared to the young adult group which had the lowest percentage of daily users at 55.2% (n =111). Kruskal-Wallis test highlighted the young adult age group as having significantly higher means than the middle-aged and older adult groups for less than daily tobacco use (mean ranks =258.15, 214.90 and 223.35 respectively). This was statistically significant (p=0.000) (Table 4).

**Table 4:** current tobacco use patterns by age cohorts

N=473	Young (n=201)	Middle-aged (n=204)	Older (n=68)	Total
Daily use	111(55.2%)	149(73.0%)	48(70.6%)	308(65.1%)
Less than Daily use	90(44.8%)	55(27.0%)	20(29.4%)	165(34.9%)
Mean Rank	258.15	214.9	223.35	

Kruskal Wallis Test X2 =16.235, df=2, p=0.000

**Perceived risk of smoking cigarettes often by age cohorts:** across all age groups, the risk perception of tobacco use does not change as most tobacco users perceived smoking cigarettes often as moderately-high. In the middle-aged and older adult age cohorts, persons with moderately high-risk perception were statistically more likely to be non-current users (p=0.008 and p=0.020 respectively). In young adults however, there was no statistical difference in current users versus non-current users with regards to perception of risk at 93% and 94.3% respectively (p=0.686) (Table 5). Three persons out of the young adult group and 2 out of the middle-aged adult group did not did not respond to sections asked on risk perception.

**Table 5:** perceived risk of smoking cigarettes often by age cohorts

	Low	Moderate/ High	Chi-Square
**Young adults (N=413)**			X2=0.298, p=0.686
Current (n=201)	14 (7.0%)	187 (93.0%)	
Non-current (n=212)	12 (5.7%)	200 (94.3%)	
**Total**	**26 (6.3%)**	**387 (93.7%)**	
**Middle-aged adults (N=513)**			X2=8.058, p=0.008
Current (n=203)	14 (6.9%)	189 (93.1%)	
Non-current (n=310)	6 (1.9%)	304 (98.1%)	
**Total**	**20 (3.9%)**	**493 (96.1%)**	
**Older adults (N=210)**			X2=7.079, p=0.020
Current (n=67)	8 (11.9%)	59 (88.1%)	
Non-current (n=143)	4 (2.8%)	139 (97.2%)	
**Total**	**12 (5.7%)**	**198 (94.3%)**	

**Main predictors for current tobacco use in young adults:** regression analysis of risk perception and selected sociodemographic factors in young adults revealed that only gender was significantly associated with current tobacco use. For young adults, gender was the main significant predictor. Given the combination of these variables, the model explains 9.7% variance in the dependent variable, which showed that being female gender was a protective factor, as males were 2.565 times more likely to be current tobacco users than females (r=0.942, OR=2.565, p<0.01) (Table 6).

**Table 6:** multivariate logistic regression analysis for current tobacco use in adults

	Young adults			Middle-aged adults			Older adults		
Co-variates	Regression Coefficient	OR	P-value	Regression Coefficient	OR	P-value	Regression Coefficient	OR	P-value
Male Gender	0.942	2.565	0.000	-0.781	0.458	0.001	0.414	1.512	0.321
Marital Status (Single)	0.310	1.363	0.191	0.516	1.675	0.003	0.590	1.803	0.026
Education (Primary level or below)	0.130	1.139	0.540	0.460	1.584	0.014	0.013	1.013	0.961
Employment	-0.138	0.871	0.507	-0.056	0.945	0.814	0.527	1.693	0.148
Occupation description	-0.344	0.709	0.057	-0.125	0.882	0.413	-0.316	0.729	0.265
Risk perception sometimes (Low)	0.405	1.499	0.156	0.471	1.602	0.091	1.035	2.815	0.025
Risk perception often (Low)	0.001	1.001	0.998	1.217	3.375	0.044	0.577	1.781	0.452
	Nagelkerke R2=0.097, Cox and Snell 0.073, Deviance=540.968			Nagelkerke R2=0.121, Cox and Snell 0.090, Deviance=640.438			Nagelkerke R2=0.147, Cox and Snell 0.05, Deviance=239.753		

**Main predictors for current tobacco use in middle-aged adults:** regression analysis of risk perception and selected sociodemographic factors in middle-aged adults revealed that gender, marital status, education and risk perception of smoking often, were significantly associated with current tobacco use. This combination of variables accounted for 12.1% variance in the dependent variable. Male gender was 0.458 times more likely to be current tobacco users (r=-0.781, OR=0.458, p=0.001). Being single was 1.675 times more likely to be current tobacco users than those who were married or divorced (r=0.516, OR=1.675, p=0.003). Those with lower education were 1.584 times more likely to be current users, than those who had higher educational attainment (r=0.460, OR=1.584, p=0.014). Additionally, those with lower risk perception to smoking cigarettes often were 3.375 times more likely to be current tobacco users than those with high risk perception (r=1.217, OR=3.375, P=0.044) (Table 6).

**Main predictors for current tobacco use in older adults:** regression analysis of risk perception and selected sociodemographic factors in older adults revealed that risk perception of smoking cigarettes sometimes and marital status, were significantly associated with current tobacco use. This combination of variables accounted for variance of 14.7% in the dependent variable. Single persons were 1.803 times more likely to be current tobacco users, as compared to being married or divorced (r=0.590, OR=1.803, p=0.026). Individuals having a low risk perception to smoking cigarettes sometimes were 2.815 times more likely to be current users, than those with moderate to high risk perception (Table 6).

## Discussion

Previous studies on tobacco use have focused on either adolescent population only or adolescent and adult populations. Lifetime and current prevalence of tobacco use amongst adolescents in Jamaica´s most recent school survey in 2013, was 27.5% and 4.4% respectively [36]. According to the Pan American Health Organization, a subsequent study in 2015, reported that current tobacco use was approximately 17% amongst Jamaican adolescents and adults [14]. This is the first study in Jamaica and the Caribbean that has focused on tobacco use solely in adults. In this study, the lifetime prevalence of tobacco smoking in the adult population was 28%. The highest prevalence was seen in the older adult category at 37%, followed by the middle aged and young adult groups at 30% and 23% respectively. The prevalence of current tobacco use in the adult population was 41.5%. Interestingly, the prevalence of current tobacco use was highest amongst the young adult population, and seen to progressively decrease as the groups advanced in age. Collectively, these studies highlight a higher preponderance of tobacco use amongst the under-researched adult population, further reinforcing the importance in providing a focused discussion regarding intervention for this group. The higher prevalence of non-current use in middle-aged and older adults, suggests that at least 2 out of every 3 users in the age group were either former users in partial, or full remission, or experimented at a younger age. The anti-smoking campaigns and policies implemented by the Government of Jamaica, as well as heavy taxation on cigarettes may have contributed to this higher prevalence of non-current use in the older age groups [14]. However, those individuals who were current users in the older age groups, were more likely to be daily tobacco smokers. This pattern of usage is more indicative of dependency as these persons are less likely to successfully discontinue without individualized intervention programs. Nelson *et al*. further reinforce this point, highlighting that daily smoking is highly resistant to change despite self-motivation and multiple quit attempts [37].

Furthermore, the increased current usage in young adults has far-reaching implications for public health policies as tobacco smoking contributes to the nation´s chronic disease epidemic that is likely to exacerbate the disease burden with advancing age. Hair *et al*. emphasize that significant intervention before age 21 most likely hinders the progression into a substance use disorder and other long-term effects [38]. For this study, the risk perception emphasis was focused on the smoking of cigarettes sometimes and often. The majority of current users reported a moderately-high risk perception towards smoking cigarettes often. For both the middle-aged and older adults, there was a significant difference between current and non-current users based on their risk perception, of at least a 5%, and 9%-point difference respectively (p=0.008 and p=0.020 respectively). However, this finding did not find replication amongst the young adult age group as there was no significant difference regarding risk perception between current and non-current users (p=0.686). This finding was further strengthened from the multivariate analysis for current tobacco use, which indicated that the perceived risk of smoking cigarettes sometimes or often amongst young adults, was not statistically significant (p=0.156 and p=0.998 respectively). This highlights a paradoxical picture. Current users know the harm associated with smoking, but nonetheless continue to smoke. Research indicates that this may be attributable to an unrealistic “optimism bias” by the current users wherein they acknowledge the associated risk in others, albeit minimizing the severity of their own risk [39]. Logistic regression model indicated that young adult males were 2.565 times more likely to be current tobacco users than females (r=0.942, OR=2.565, p<0.01). This association is not farfetched as males are more inclined to be risk-takers, engage in unhealthy behaviors, and be less forthcoming in seeking help when warranted [40]. Furthermore, the behavioral patterns are reinforced during the vulnerable young adulthood phase of development [41].

Logistic regression model indicated a similar finding amongst middle-aged adult males (r=-0.781, OR 0.458, P=0.001). Additionally, the model indicated that single middle-aged adults were 1.675 times more likely to be current tobacco users than those who were married or divorced (r=0.516, OR=1.675, p=0.003) and that those with lower education were 1.584 times more likely to be current users, than those who had higher educational attainment (r=0.460, OR=1.584, p=0.014). Literature has highlighted both the elevated risk associated with single status and the protective role of marriage on tobacco use [42,43]. Moreover, individuals with high school or lower education achievement were more likely to be current smokers [44]. In furtherance, the model indicated that middle-aged adults with lower risk perception to smoking cigarettes often were 3.375 times more likely to be current tobacco users those with high risk perception (r=1.217, OR=3.375, P=0.044). Extant literature suggests that moderate to high risk perception increases the likelihood of quit attempts and smoking cessation [33]. In older adults, logistic regression model indicated a similar finding in that single individuals were 1.803 times more likely to be current tobacco users, compared to those being married or divorced (r=0.590, OR=1.803, p=0.026). This finding is supported by literature that suggests marriage motivates positive and healthy behavior [43]. However, this partnership must be supportive, as having a partner that fails to emphasize healthy lifestyle behaviors, can hinder a positive change especially in older age [45]. Additionally, the model indicated that older adults with a low risk perception to smoking cigarettes sometimes, were 2.815 times more likely to be current users than those with moderate to high risk perception (r=1.035, OR=2.815, p=0.025).

Evidently, risk perception played a significant protective role in all age groups except for young adults. This indicates that the strategies for tobacco smoking cessation geared towards health education and increased risk awareness, may not be as singularly effective in young adults, and may require more modern and unique approaches. Furthermore, in recognizing, that 4 out of 10 Jamaican adult tobacco users are current users, strategies geared towards the fight against tobacco use is paramount. On a collective level, policymakers could introduce heavier taxation and restructuring of the applied rates on all tobacco products, especially cigarettes; and heavier fines and legal ramifications for importers who are involved in the distribution of the burgeoning illicit, counterfeit tobacco products trade [46-48]. Currently, there is a lack of national smoking cessation clinics on offer or subsidized nicotine replacement therapeutic options, that have found support in increasing quit attempts [49]. On an individual level, career incentives by way of job recruitment endorsing a “no smoking” criteria for employment, could encourage prospective employees to abstain from smoking [50]. In addition, strong evidence for a lifestyle changing strategy is supported by an American study that highlighted decreased cigarette use when physical activity was incorporated [51]. There were some limitations to this study. The study utilized a cross-sectional design and thus only associations could be determined and not causal relationships. In addition, the household survey only contained respondents from households and as such excluded a percentage of the population who were homeless, in prisons, and health care institutions. Also, this study utilized a self-report survey related to substance use behavior, and therefore findings may suffer from underreporting bias.

## Conclusion

The present study suggests that risk perception in young adults had no significant influence on current tobacco use despite high awareness. Furthermore, the prevalence of current use in young adults was highest. While health promotion and education have been effective in increasing the risk perception amongst middle-aged and older adult groups, this study indicates more innovative and contemporary approaches are needed for the younger cohort. The sociodemographic associations also highlight the extent of tobacco misuse, and reemphasize the need for targeted rehabilitation programs and support groups, as the individual transitions from young to older adulthood. There are a few insights gleaned from this study that can be of significant contribution for further research with regards to tobacco use in Jamaica. An analysis of female tobacco users compared to their counterparts will assist in creating a profile of tobacco users based on gender. This will aid policymakers in a targeted approach to interventions based on the gender group. Additionally, exploring tobacco use amongst adults when combined with other drugs could be beneficial, especially since multiple drug use has a higher morbidity and mortality than single substance use. Lastly, since Jamaica signed onto the WHOFTC in 2005 and implemented new policies surrounding tobacco use, further study would prove useful in elucidating whether the control measures are being utilized and to what extent, since the current use prevalence remains relatively high.

### What is known about this topic

- Tobacco smoking has the highest associated mortality and morbidity when compared to other substances;
- The lifetime prevalence of tobacco use is gradually decreasing globally, more so in men than women;
- Smoking cessation is quite challenging, with higher percentage of quit attempts, but much lower successful quitters.

### What this study adds

- The young adult population has the highest current tobacco use prevalence compared to middle-aged, and older adult population;
- Risk perception of tobacco smoking in young adults presents a paradoxical picture; young adults perceive high risk with smoking tobacco, yet have the highest current use prevalence;
- This study highlights the urgent need for more targeted preventative and rehabilitative programs within each adult group, with earliest intervention in young adults.
